# Prioritizing functional phosphorylation sites based on multiple feature integration

**DOI:** 10.1038/srep24735

**Published:** 2016-04-19

**Authors:** Qingyu Xiao, Benpeng Miao, Jie Bi, Zhen Wang, Yixue Li

**Affiliations:** 1 Key Lab of Computational Biology, CAS-MPG Partner Institute for Computational Biology, Shanghai Institutes for Biological Sciences, Chinese Academy of Sciences, Shanghai, P. R. China; 2 University of Chinese Academy of Sciences, Beijing, P. R. China; 3 Key Lab of Systems Biology, Institute of Biochemistry and Cell Biology, Shanghai Institutes for Biological Sciences, Chinese Academy of Sciences, Shanghai, P. R. China; 4 Shanghai Center for Bioinformation Technology, Shanghai Industrial Technology Institute, Shanghai, P. R. China

## Abstract

Protein phosphorylation is an important type of post-translational modification that is involved in a variety of biological activities. Most phosphorylation events occur on serine, threonine and tyrosine residues in eukaryotes. In recent years, many phosphorylation sites have been identified as a result of advances in mass-spectrometric techniques. However, a large percentage of phosphorylation sites may be non-functional. Systematically prioritizing functional sites from a large number of phosphorylation sites will be increasingly important for the study of their biological roles. This study focused on exploring the intrinsic features of functional phosphorylation sites to predict whether a phosphosite is likely to be functional. We found significant differences in the distribution of evolutionary conservation, kinase association, disorder score, and secondary structure between known functional and background phosphorylation datasets. We built four different types of classifiers based on the most representative features and found that their performances were similar. We also prioritized 213,837 human phosphorylation sites from a variety of phosphorylation databases, which will be helpful for subsequent functional studies. All predicted results are available for query and download on our website (Predict Functional Phosphosites, PFP, http://pfp.biosino.org/).

Protein phosphorylation is an important type of post-translational modification that is involved in a variety of biological activities, including molecular association, protein degradation, enzymatic activation/inhibition and intracellular localization^1^,^2^,^3^. The phosphorylation events usually occur on serine (S), threonine (T), tyrosine (Y), histidine (H) and aspartic acid (D) residues in eukaryotes^4^,^5^, and we mainly focused on S, T and Y due to their prevalence in nature. A phosphate (PO4−) group can be either added by kinases or removed by phosphatases, resulting in a reversible molecular switch^6^. It has been reported that aberrant phosphorylation is associated with many human diseases such as Alzheimer’s disease, diabetes and cancer^7^,^8^,^9^.

A large number of phosphorylation sites (phosphosites) have been identified as a result of advances in mass spectrometric techniques in recent years, which provides great opportunities and challenges for functional studies^10^,^11^,^12^. However, only a small number of phosphosites have characterized functions, and the functional roles of many phosphosites remain unclear. Landry *et al.* studied the phosphoproteomes from an evolutionary perspective and suggested that a large percentage of phosphosites were non-functional (the estimated proportion was 65%)^13^,^14^. It was proposed that the non-functional phosphosites may result from the off-target recognition of kinases^13^. Phosphosites with important functions are of great value for both experimental and computational biology. However, conventional predictors concentrate on predicting whether a site is phosphorylated, and few make any prediction as to whether the site itself is functional^15^,^16^,^17^. Furthermore, with the high-throughput rate of mass spectrometric analyses of protein phosphorylation, there is likely a high proportion of non-functional sites and issues with localization accuracy^18^,^19^,^20^. Studies of non-functional sites are often unproductive or yield wholly misleading results. Therefore, the identification of functional phosphosites must be addressed.

Several studies have aimed to identify functional phosphosites using computational strategies. Niu *et al.* calculated both absolute and relative conservation scores to predict the most likely functional phosphosites^21^. However, conservation alone is not sufficient for measuring the function of a site, as some functional phosphosites are not conserved at specific positions^22^,^23^. Beltrao *et al.* prioritized the functional relevance of post-translational modifications by predicting sites that were likely to be involved in cross-regulatory events, domain activity regulation, or protein-protein interactions^24^. Their study highlighted the importance of using multiple sources of information to characterize functional phosphosites. However, a systematic method of integrating and evaluating the features for functional phosphosite identification was not provided. The current study focused on evaluating the intrinsic characteristics of functional phosphosites and predicting whether a phosphosite is likely to be functional using multiple integrated characteristics. Features including conservation, kinase association and structure information were explored and compared in both known functional and background phosphosite datasets. The two datasets were classified by several trained classifiers based on feature integration. We also used the model to screen all human phosphosites collected from a variety of databases to provide the most likely candidates for further study.

## Results

### Training set

The construction of training datasets is critical for machine learning analysis. In this study, known functional human phosphosites from the PhosphoSitePlus database (file name: Regulatory_sites, version: 060415) were used as the positive dataset^25^. For the negative data, we first filtered all human phosphosites to high-quality data subsets according to the counts of low/high-throughput experiments (see Methods). It is difficult to conclude that a phosphosite is non-functional in practice. A feasible solution is to find “background phosphosites” that are polymorphic in the human population and are not associated with diseases (see Methods). We also removed phosphosites on redundant proteins (sequence identity >30%) in both datasets to avoid possible bias. Finally, we preserved 3,557 and 1,943 phosphosites in the positive and negative datasets, respectively (see Methods; Supplementary Figure 1). To balance the two datasets, we removed the sites that overlapped with the NetworkIN training data. Then, we randomly chose 1,911 sites from each dataset while matching S/T/Y and the number of homologous peptides. As a result, for both the positive and the negative datasets, the proportion of S/T/Y phosphosites is 58.87%, 24.02% and 17.11%, respectively, and the distributions of the number of homologous proteins in humans for the two datasets are the same (Supplementary Figure 2).

We analyzed the constructed positive dataset to validate the rationality of the “polymorphic and not associated with diseases” rule. Among the 82,940 high-quality phosphosites, 3,103 sites were marked “negative” according to the rule, while 152 of 5,372 known functional sites were marked “negative”. We tested the two proportions, and the result revealed a significant difference (p-value = 0.001, Chi-squared test).

### Evolutionary conservation

**Figure 1:**
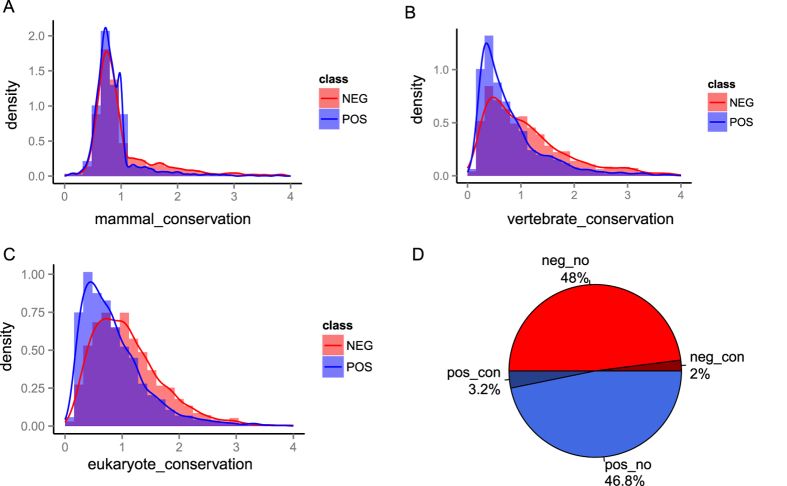
The conservation of phosphosites. The distributions of sequence conservation for three different groups: (**A**) mammal group, (**B**) vertebrate group, and (**C**) eukaryote group. For each group, the distribution of the positive dataset is significantly different from the distribution of the negative dataset (p-value < 2.2e-16, t-test). (**D**) The distribution of status conservation. The positive and negative datasets exhibit significant differences in status conservation (p-value = 4.74e-4, chi-squared test). pos: positive dataset; neg: negative dataset; con: conserved phosphosites; no: non-conserved phosphosites.

It was previously reported that phosphosites with known function are more likely to be conserved than those with no characterized function^13^,^26^,^27^,^28^. If some phosphosites were more conserved in evolution, we could infer that these sites have potential function^29^. In this study, we investigated both the sequence and the status conservation of phosphosites^24^. The sequence conservation of a phosphosite was evaluated by its evolutionary rate across 14 eukaryotes. In general, the faster the evolutionary rate a phosphosite has, the less conserved it is. Although this method has been widely used to score conservation^13^,^30^,^31^, variation in the evolutionary rate was not taken into account. To avoid this problem, we divided the 14 species into three groups (mammal, vertebrate and eukaryote), which represented close, median and distant time scales in the evolutionary tree, respectively. We calculated the evolutionary rate for each group separately (see Methods). Although the three evolutionary rates differed among the groups (Supplementary Figure 3), they were all significantly lower in the positive dataset than in the negative dataset (mammal: p-value < 2.2e-16; vertebrate: p-value < 2.2e-16; and eukaryote: p-value < 2.2e-16, t-test, Fig. 1A–C), suggesting they could be used for scoring functional phosphosites.

As most phosphosites are located in disordered regions with a fast evolutionary rate, some functional phosphosites may have poor sequence conservation^13^. In addition, the precise positions of phosphosites are not necessary for the regulation of phosphorylation, particularly in disordered regions^22^,^23^,^24^,^32^,^33^. To account for this, we defined the status conservation of phosphosites, which only requires its homologous sites to be phosphorylated nearby^24^. As there are fewer phosphosites in other species than in humans, we investigated the status conservation of phosphosites by comparing the homologous peptides only within the human genome. To select the proper parameters, we considered a wide range of homologous peptide lengths (5~14) and phosphosite positions (±1~10) (see Methods, Supplementary Tables 1 and 2). We found that a peptide length of 11 and a position of ±5 were the best parameters to evaluate the status conservation because they led to the most significant differences between the positive and negative datasets (p-value = 4.74e-4, Chi-squared test, Fig. 1D). These results indicate that both the sequence and the status conservation could contribute to functional phosphosite screening.

### Structural environment

Protein properties and functions are highly correlated with their structure. Disordered regions often do not have well-folded persistent structures and are enriched for protein-protein interactions because of their variability for flexible conformational change^34^,^35^,^36^. Due to their easier accessibility to kinases, most phosphosites occur in disordered regions, which are less conserved than ordered regions^16^,^37^. Thus, in such regions, the off-target effects of kinases may occur more frequently, which could result in a large number of non-functional phosphosites. In addition to the disorder state, the underlying secondary structure may also have a great impact on the function of a phosphosite^38^,^39^. In this study, for each phosphosite, we calculated the disorder score and the types of secondary structures, i.e., beta sheet, helix and coil.

**Figure 2:**
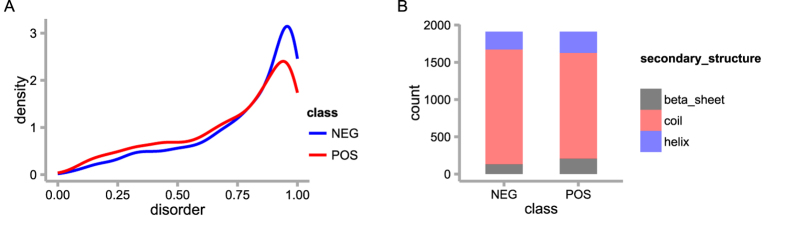
The distribution of structural features in the positive and negative datasets. (**A**) Disorder score distribution (p-value < 2.2e-16, Wilcoxon rank sum test). (**B**) Secondary structure distribution (p-value = 2.29e-6, chi-squared test).

To evaluate the predictive ability of each feature, we compared their distributions in the positive and negative datasets. In both datasets, the majority of phosphosites are located in the disordered region (Fig. 2A). However, more sites in the positive dataset have relatively lower disorder scores, indicating that more positive sites are located in ordered regions compared to negative sites. The score distributions in the two datasets are significantly different (p-value < 2.2e-16, Wilcoxon rank sum test). This result is consistent with our expectation that background phosphosites occur more frequently in disordered regions, where more off-target events could arise.

We compared the distribution of secondary structures in the two datasets and found that they were also significantly different (p-value = 2.29e-6, Chi-squared test). As shown in Fig. 2B, most of the phosphosites in both datasets prefer to locate in coils, but the proportion of sites in coil structures in the negative dataset is significantly higher than that in the positive dataset, which is in agreement with the distribution of disorder scores.

### Association with kinase

**Figure 3:**
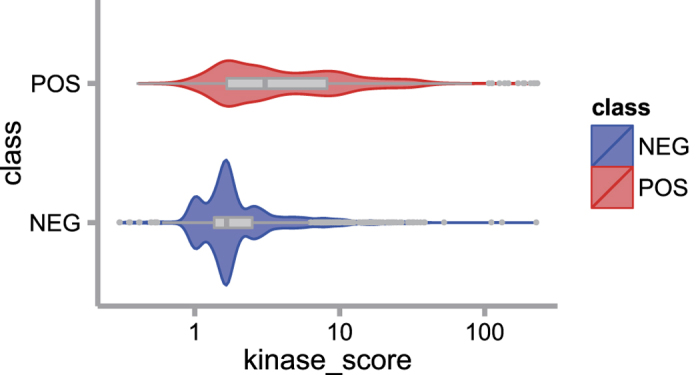
NetworkIN score distribution. Violin plot for NetworkIN score with log-scaled x-axis. The figure shows the significant difference between positive and negative sites (p-value < 2.2e-16, Wilcoxon rank sum test).

Due to the off-target activity of kinases, we hypothesized that randomly phosphorylated sites tended to have weaker associations with kinases^13^. If a phosphosite is functional, it is more likely to match the kinase motif perfectly. Therefore, measuring the association between kinases and phosphosites may help identify those sites with critical functions. NetworkIN is a tool that integrates both protein-protein interaction and phylogenetic tree information to predict kinase recognition, and it returns a NetworkIN score for each site in a probabilistic manner^40^. A larger NetworkIN score indicates a higher possibility of kinase recognition. We compared the distribution of NetworkIN scores in the two datasets (see Methods). As shown in Fig. 3, positive sites have significantly higher NetworkIN scores than negative sites (p-value < 2.2e-16, Wilcoxon rank sum test). This result indicates that kinase association is a good feature to distinguish positive sites from negative ones.

### Different model performance

To find the most important and robust features for model building, we performed a 50-round feature selection procedure. For each round, we generated a randomly chosen balanced dataset and selected an optimal subset from the features investigated above using the attribute selection tools in Weka (see Methods)^41^. Among the features, some were very robust (e.g., kinase score was selected as the best feature in all 50 rounds). We counted the frequency of each feature as it appeared in the selected feature subset and chose the top 5 high-ranked features. Consequently, the final robust feature subset included the kinase score, sequence conservation in mammals and eukaryotes, disorder score, and beta-sheet state.

**Figure 4:**
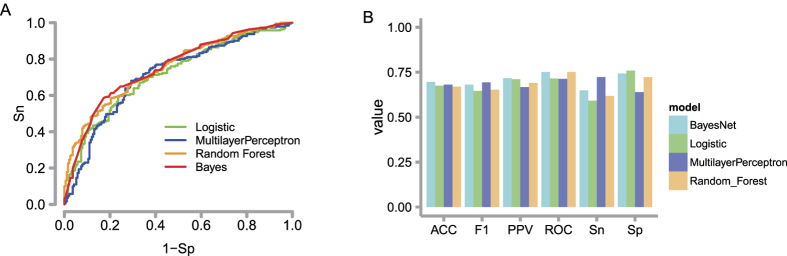
Different model performance on the independent test set. (**A**) The ROC curve for different models. Different models yield comparable results. (**B**) Sensitivity, specificity, accuracy, precision, F-measure and the area under the ROC curve for five models. Sn: sensitivity; Sp: specificity; ACC: accuracy; PPV: precision; F1: F-measure; and ROC: the area under the ROC curve.

We trained four different types of machine learning models, including Bayesnet, Logistic regression, Random Forest and Multilayer Perceptron. We split the full datasets into 90% for the training set and 10% for the independent test set. With the 10-fold cross-validation method performed on the training set (see Methods), we compared the performance of the four models (Supplementary Figure 4). The results indicate that the performances of the constructed models are similar and robust. Figure 4A shows the ROC curve of the independent testing datasets for different models, and different models yield comparable results, which indicate that the prediction is independent of the model chosen. The detailed performance metrics of the independent test set including sensitivity, specificity, precision, F-measure, accuracy and the area under the ROC curve for the four models are compared in Fig. 4B. The performances on the test dataset are similar to the cross-validation results on the training dataset, indicating no overfitting of the model.

To evaluate the relative contribution of the features, we assigned the five selected features into 3 groups: kinase association (kinase score), sequence conservation (eukaryote conservation and mammal conservation), and structure (disorder score and beta-sheet state). We used each group alone to build the model with the Random Forest. Comparing the ROC curves of the 3 feature groups (Supplementary Figure 5), we found that kinase association played the most important role, and sequence conservation played the second most important role.

### Prediction for the whole dataset

**Figure 5:**
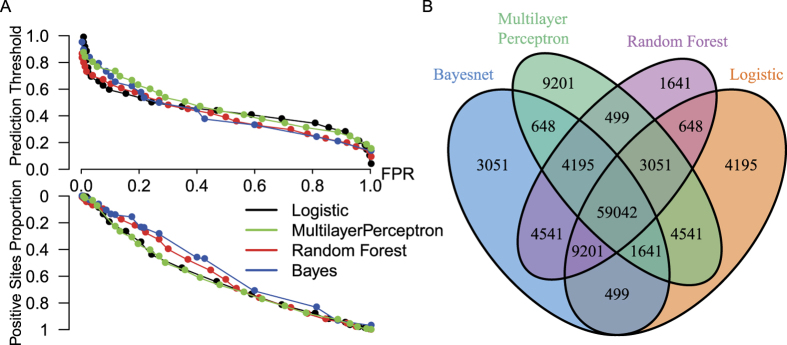
Evaluation of the prediction results of the different models. (**A**) The upper half of the figure shows the relationship between the threshold of prediction score and false-positive rate (FPR). The lower half of the figure shows the relationship between the proportion of predicted positive sites and FPR. The results indicate that a larger threshold of prediction score is often associated with a lower false-positive rate and proportion of predicted positive sites. (**B**) The Venn diagram of the prediction results of the different models (FPR = 0.1) for all human phosphosites (213,837). The results of the different models show good accordance.

We applied all four models to the whole human phosphorylation dataset collected from the PhosphositePlus, PhosphoELM, and dbPTM databases^42^,^43^. For each site predicted, the model yields a prediction score ranging from 0 to 1, indicating the extent of the prediction to be positive. As different thresholds of the prediction score would give different proportions of predicted positive/negative sites, we showed the relationship between the false-positive rate and the threshold, as well as the predicted proportion of positive sites for different models (Fig. 5A).

We predicted a total of 213,837 phosphosites on 19,247 proteins and compared the prediction results of the different models (false positive rate = 0.10) as shown in Fig. 5B and Supplementary Figure 6. Approximately 68.7% of all phosphosites have the same prediction results for the four models, while 93.0% have consistent prediction results for at least three models (Fig. 5B). When limited to the high-quality data, the above proportions become 71.3% and 93.1% for the four models and for at least three models, respectively (Supplementary Figure 6). These results show that the predictions of the different models are in good accordance and emphasize the value of combined use of the results of the different models as well as a proper threshold of prediction score for credible screening.

All data are available for query and download on our PFP website (Predict Functional Phosphosites, PFP, http://pfp.biosino.org/). Users can use the UniProt ID (or HGNC gene symbol) and the position information of the phosphosite to query whether the site is more likely to be functional for subsequent experimental validation. To make it easier for users to compare the different models and to select their own threshold, the prediction results and prediction scores for all four models are also available.

We also used the predicted results on high-quality phosphosites to validate whether the “polymorphic but not associated with diseases” rule for the negative sites was reasonable (Supplementary Table 3). Looking at the Random Forest prediction results (false positive rate = 0.1) as an example, for the predicted 13,883 positive sites, there were only 499 sites that passed the rule, and the proportion was significantly lower than that in all phosphosites (p-value = 1.12e-4, Chi-squared test). Similarly, the proportion is also significant for the other three models (Supplementary Table 3). The results suggest that the rule is reasonable for the negative dataset construction.

## Discussion

Finding functional phosphosites from a large number of candidates will be more and more important for the study of their biological function. In this study, we systematically evaluated and integrated multiple features for functional phosphosite identification. We investigated the sequence conservation, status conservation, kinase association, disorder score and secondary structure of phosphosites. We found that all these features show significant differences between functional and background phosphosites. We selected the most representative features to build classifiers based on different algorithms, and different models performed comparably. The predicted results, along with the corresponding prediction scores, are available on our website. These results improve our understanding of the mechanism of phosphorylation and will be helpful for subsequent functional studies.

Among our optimal features, the kinase score played the most important role. The functional phosphosites have significantly stronger associations with corresponding kinases than the background sites, which provides evidence for the hypothesis that non-functional phosphosites may result from the off-target recognition of kinases^13^. In protein interactomes, there are many “noisy interactions” with no apparent functional meaning^44^. Similarly, random phosphorylation events resulting from misrecognition of kinases were suggested to be common in kinase-substrate networks^14^.

The evolutionary rate has widely been used to score conservation for evaluating the functional importance of phosphosites^13^,^21^,^24^. However, the fact that the evolutionary rate was variable in evolution was not accounted for. In this study, we considered the variation in the evolutionary rate by assigning the species into different groups. The sequence conservations of both eukaryotes and mammals were chosen as the optimal features, suggesting that it was reasonable to incorporate different levels of conservation for function evaluation.

Our results indicated that both the disorder score and the secondary structure contributed to prioritization of the functional phosphosites. Phosphosites were enriched in disordered regions for more efficient kinase recognition^13^,^16^,^38^. However, our study found the proportion of background phosphosites in disordered regions is higher than that of functional ones in disordered regions. This may result from the possibility that the off-target events of kinases are more likely to occur in disordered regions. Similarly, more functional sites are located in helix and beta-sheet structures, which are well folded compared with coils.

Although plausible in theory, non-functional phosphosites are hard to validate in practice, which is the main challenge for the construction of the negative dataset. In this study, we defined the negative dataset as the “background phosphosites” that satisfy the rule “sites with polymorphism but not associated with diseases”. Although phosphosites with SNPs are not always non-functional, their toleration in the population often implies that they are not important for key functions. In addition, our results demonstrated that the proportion of phosphosites passing the rule in the positive dataset (and predicted positive dataset) was significantly lower than that in the whole phosphosite dataset, which provided statistical evidence for the rationality and effectiveness of our method. However, there are still false-negative phosphosites that may affect the performance of our prediction. A better solution would be to find phosphosites with high-frequency polymorphisms in the human population, but these are limited due to the lack of frequency information of current SNP data. Further extension of SNP frequency information may improve the prediction ability.

## Methods

### Data collection

We downloaded all human phosphosites and regulatory phosphosites from the PhosphoSitePlus database (version: 060415)^25^. Additionally, phosphosites from dbPTM, which integrated Phospho.ELM (release 9.0), UniProtKB (release 2015-08), HPRD (release 9.0) and SysPTM (release 2.0), were also collected to build a comprehensive human phosphosite database^42^,^43^,^45^,^46^,^47^. The PhosphositePlus database provided a file named ‘Regulatory_sites.gz’ (http://www.phosphosite.org/downloads/Regulatory_sites.gz), which listed the functional annotations for phosphosites as “on known function”, “on process”, “on protein interaction” and “on other interactions”. In our study, a phosphosite is considered “known functional” if it has functional annotations in this file. In total, we retrieved 213,837 human phosphosites, which included 5,389 known functional phosphosites as the positive dataset. For negative data, we do not have a dataset that is directly ready for use. A feasible solution is to find “background phosphosites” that are polymorphic in the human population and are not associated with diseases. Taking data quality into consideration, we obtained high-quality phosphosites based on experimental data with the following rules: a site must (1) have low throughput experimental evidence; or (2) be supported by at least 2 mass spectrometry experiments. After this filtering step, we obtained 82,940 high-quality phosphosites. Next, the CD-hit software was used to remove homologous proteins with sequence identity greater than 30%^48^. After the phosphosites lying in redundant proteins were removed, we preserved 3,557 and 50,258 phosphosites in the positive and negative datasets, respectively.

Next, for the negative sites, we annotated human SNPs from dbSNP138 using ANNOVAR (version: 20150619, hg19), and we mapped all high-quality and non-redundant human phosphosites to the results^49^. From these overlapping sites, we removed those associated with disease in the ClinVar database (35 sites), COSMIC deleterious somatic mutations (496 sites), SNPs in the GWAS Catalog (0 sites), and disease-associated sites provided in the PhosphoSitePlus database (7 sites)^50^,^51^,^52^. Previous studies have indicated that SNPs influencing phosphorylation might have some functional implications^53^,^54^; therefore, we further filtered the phosphorylated SNP sites defined by PhosSNPs that may have potential functional roles (312 sites removed)^55^. After removing sites that overlapped with the positive dataset (70 sites), we obtained a negative dataset containing 1,943 background phosphosites. The overview of the data processing workflow is shown in Supplementary Figure 1.

At the following feature evaluation step, we used the NetworkIN score to evaluate the associations between phosphosites and their kinases. To avoid possible prediction bias caused by overlap of the training data, we removed the NetworkIN training sites from our positive datasets (980 sites removed, ~27.6%). This step resulted in a final positive dataset containing 2,577 phosphosites.

Because any differences between the positive and negative datasets will be detected by the machine learning model, it is important to balance some fundamental characteristics in the two datasets, such as the number of phosphosites, S/T/Y comparability, and the number of hit peptides when blasted in the human phosphoproteome (important for analysis of “status conservation” in the following part). Taking all these factors into consideration, we randomly chose 1,911 balanced phosphosites from both datasets to avoid possible bias in subsequent model construction.

### Conservation score

The conservation of phosphosites was analyzed by sequence conservation across different species and status conservation within the human proteome^24^. For sequence conservation, we first divided the 14 species into three groups: mammal (*H. sapiens, P. troglodytes, B. taurus, R. norvergicus,* and *M. musculus*), vertebrate (*H. sapiens, P. troglodytes, B. taurus, G. gallus, X. tropicalis, D. rerio, R. norvergicus,* and *M. musculus*) and eukaryote (*H. sapiens, P. troglodytes, B. taurus, G. gallus, X. tropicalis, D. rerio, R. norvergicus, M. musculus, A. thaliana, O. sativa, S. cerevisiae, S. pombe, D. melanogaster,* and *C. elegans*). Then, we found the orthologous proteins of these species in each group with human proteins, and we performed a multiple sequence alignment with Clustal-omega^56^. Finally, we used the rate4site software to calculate the evolutionary rate of each human phosphosite in the three groups, respectively^57^.

For status conservation, all the human phosphorylated proteins were used as a library. Here, we considered the influence of two parameters: length of query peptide and window of aligned positions. We extracted 5~14 residues on each side of human phosphosites as the query peptide to perform a blast against the library^58^. For a phosphosite, if its corresponding homologous peptide was also phosphorylated within a window around the aligned positions, it was considered to have status conservation, regardless of the type of residues phosphorylated^24^. Here, we considered a wide range of window size (±1~10). By evaluating the differences in the positive and negative datasets, we chose 11 as the query peptide length and ±5 around aligned positions to calculate the status conservation (Supplementary Tables 1 and 2).

### Disorder score and secondary structure

The VSL2 software integrates 26 sequence-based features to predict intrinsically disordered regions on given protein sequences^59^. It calculates the disorder score (between 0 and 1) for each amino acid. The higher the disorder score, the more likely the corresponding amino acid lies in a disordered region. SSpro/ACCpro (release 5.1) software is widely used for predicting the secondary structure of given proteins^60^. SSpro predicts 3 classes of secondary structures, including beta sheet, helix and coil. In this study, we separately coded these 3 classes by “001”, “010” and “100”. To effectively run the software, we extracted 40 amino acids around each phosphosite (i.e., peptide length = 81).

### NetworkIN score

NetworkIN is a convenient tool that predicts the most likely kinase that catalyzes the given phosphosites^40^. NetworkIN uses various types of experimental data, such as position-specific scoring matrices (PSSMs) generated by peptide array experiments and *in vivo* phosphatase interactions. Moreover, relationships between individual kinases and their specific downstream phospho-proteins were manually curated from the literature, where the specificity of the relationships has been carefully tested and validated^40^. The algorithm integrates STRING-derived proximity scores and NetPhorest probabilities as the final NetworkIN score, which represents how likely the phosphorylation interaction is to occur^40^. Because of the probabilistic manner of the NetworkIN score, we can directly compare predictions from different kinases without normalization^40^. For each phosphosite, we set the maximum NetworkIN score as the “NetworkIN feature”, indicating the association strength between a specific phosphosite and its corresponding kinases.

### Feature selection

We used the attribute evaluator CfsSubsetEval in Weka (version 3.6)^41^ and set the search method as BestFirst. CfsSubsetEval takes the predictive ability of each feature and the redundancy among different features into consideration, and it selects the best subsets of features that have high correlations with the class and low correlations among the features. The BestFirst method combines a greedy hill-climbing algorithm with a backtracking facility.

To evaluate the robustness of the selected features, we randomly chose the balanced datasets for 50 rounds. For each round, we used a 10-fold cross-validation method during feature selection, and we selected the feature subsets that could best classify the two datasets. The most robust features in 50 rounds were selected as our final feature subset and were used in the following model construction.

### Training and prediction

Weka integrates many different types of machine learning methods^41^. We used four representative classifiers (Bayesnet, Logistic, Random Forest and Multilayer Perceptron) to build models, and we compared their performance. For each model, we performed parameter optimization using Weka’s meta-classifier CVParameterSelection, which searches for the best parameter settings in a given range^41^. To address missing values, which are not allowed in some models, we replaced them with the means for numeric attributes (or modes for nominal attributes) from the training data. We split the full dataset into 90% for the training set and 10% for the independent test set (keeping S/T/Y and the numbers of homologous proteins balanced), and we repeated the training and prediction step 10 times using balanced cross-validation on the training data to evaluate model performance and robustness.

### Model evaluation

We used the 10% independent test dataset to evaluate model performance. The definitions of the performance metrics are as follows:


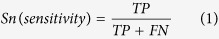



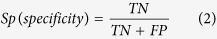



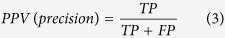



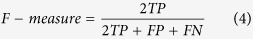



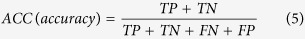



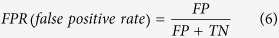


where TP, TN, FP, and FN represent the number of true positives, true negatives, false positives and false negatives, respectively. We also used receiver operating characteristic (ROC) curves to visualize the performance and compare the different models.

## Additional Information

**How to cite this article**: Xiao, Q. *et al.* Prioritizing functional phosphorylation sites based on multiple feature integration. *Sci. Rep.* **6**, 24735; doi: 10.1038/srep24735 (2016).

## Supplementary Material

Supplementary Information
