# Corrigendum: Prioritizing functional phosphorylation sites based on multiple feature integration

**DOI:** 10.1038/srep28413

**Published:** 2016-06-27

**Authors:** Qingyu Xiao, Benpeng Miao, Jie Bi, Zhen Wang, Yixue Li

Scientific Reports 6: Article number: 2473510.1038/srep24735 published online: 04192016; updated: 06272015.

The original version of this Article contained typographical errors in the Predict Functional Phosphosites website ‘http://pfp.biosino.org/pfp’ which was incorrectly given as ‘http://pfp.biosino.org/’. These errors have now been corrected in the PDF and HTML versions of the Article.

