# Supplementary material for: Prioritizing functional phosphorylation sites based on multiple feature integration
Source: Sci Rep. 2016 Apr 19;6:24735. doi: 10.1038/srep24735 (PMC4835696; doi:10.1038/srep24735)
Supplement: Supplementary Information [file srep24735-s1.pdf]

Supplementary Information

Prioritizing functional phosphorylation sites based on multiple feature integration

Qingyu Xiao1,2*, Benpeng Miao1,2*, Jie Bi1,2, Zhen Wang#1, Yixue Li#1,3

1 Key Lab of Computational Biology, CAS-MPG Partner Institute for Computational Biology, Shanghai Institutes for Biological Sciences, Chinese Academy of Sciences, Shanghai, P. R. China

2 University of Chinese Academy of Sciences, Beijing, P. R. China

3 Shanghai Center for Bioinformation Technology, Shanghai Industrial Technology Institute, Shanghai, P. R. China

* These authors contributed equally to this work.

# Correspondence should be addressed to Zhen Wang (zwang01@sibs.ac.cn) and Yixue Li (yxli@sibs.ac.cn).

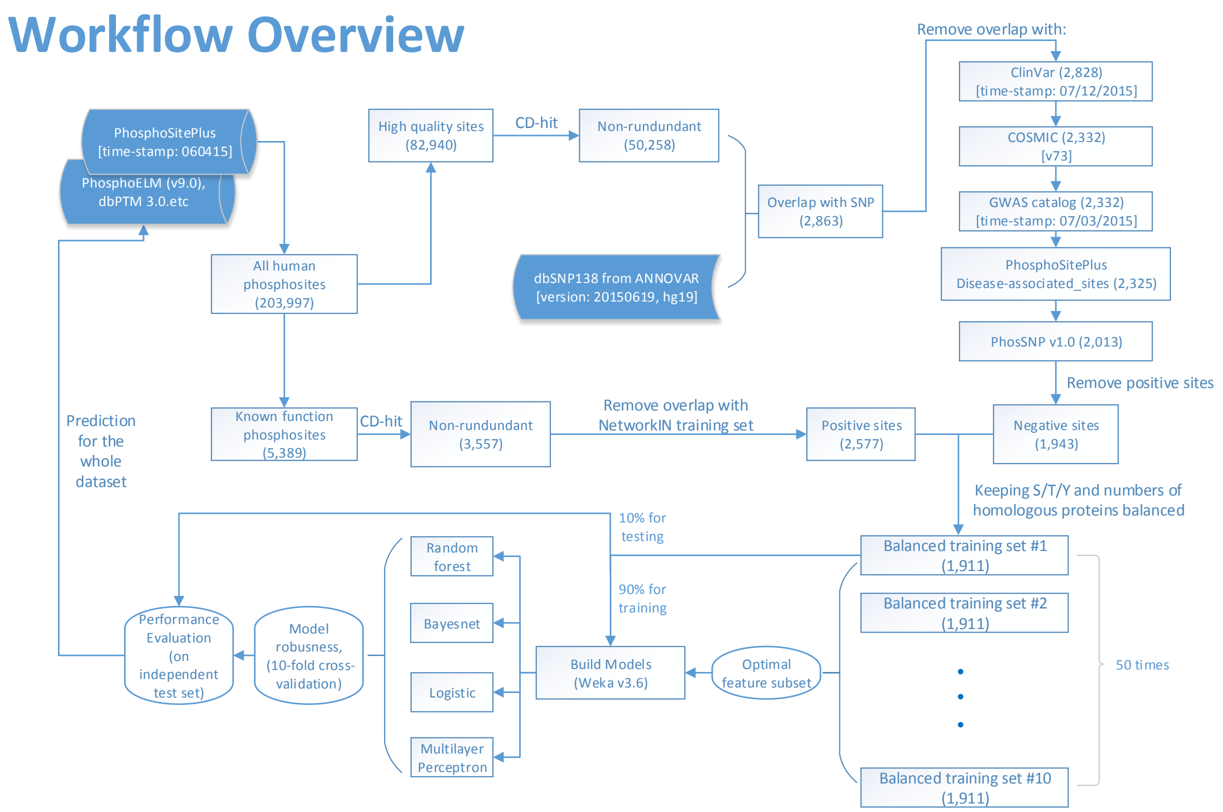

Supplementary Figure 1. The data processing workflow. The time-stamp/version of all databases downloaded and the phosphosite numbers pre- and post-filtering of each step were listed.

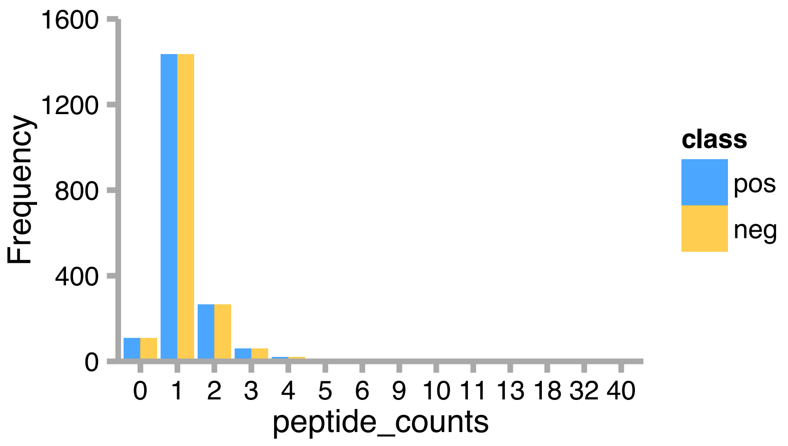

Supplementary Figure 2. Distributions of the number of homologous proteins in human for the positive and negative datasets are the same.

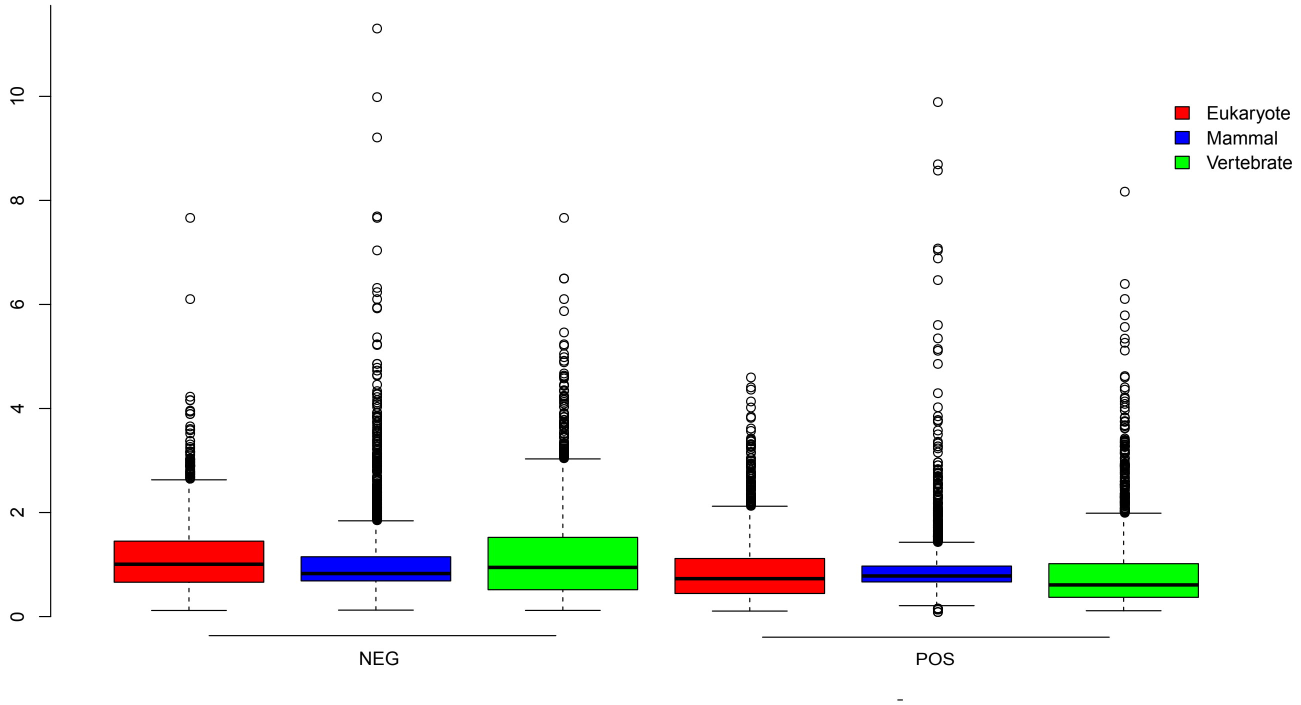

Supplementary Figure 3. The evolutionary rates of the three different groups. The three evolutionary rates were different among the groups.

POS: positive dataset; NEG: negative dataset.

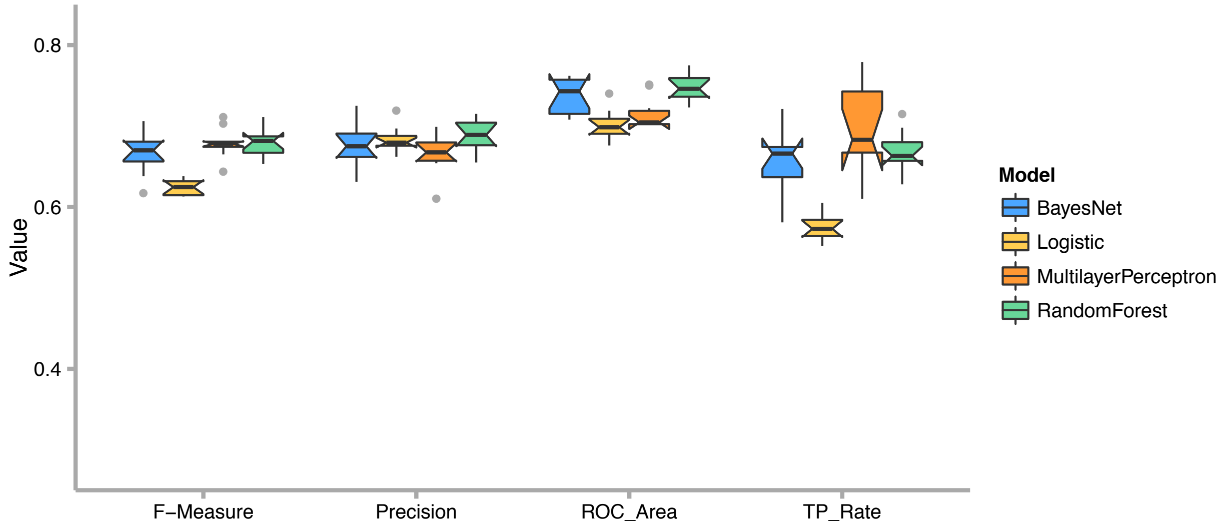

Supplementary Figure 4. The boxplot of different models' performance (F-Measure, Precision, ROC Area and TP rate) on training set during 10 times cross-validation. Notch shows the median confidence interval. Different models' performances are robust and comparable.

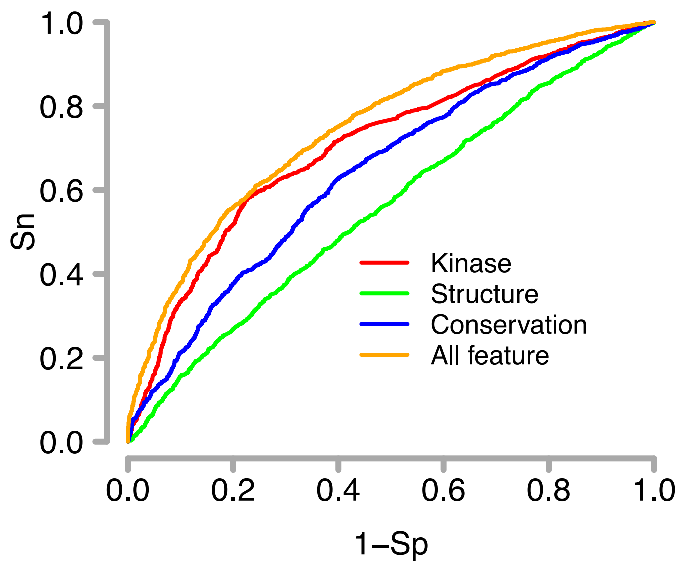

Supplementary Figure 5. The ROC curve using different feature groups. Random forest model was built based on each feature group with the same training data and parameters. From the area under ROC curve, each feature group's relative contribution/predictive ability could be evaluated. The model reached the best performance when using all features together.

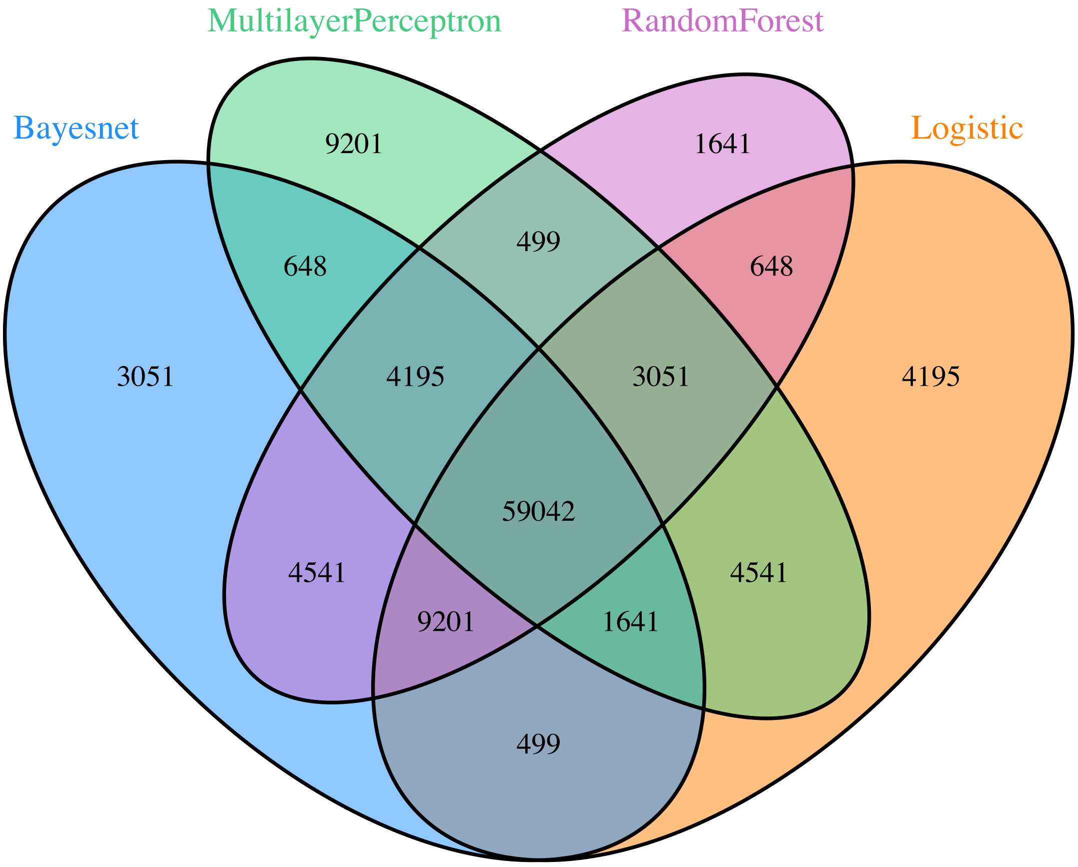

Supplementary Figure 6. The Venn diagram of different models' prediction results for high-quality phosphosites (84,818). The results of different models show good accordance.

Supplementary Table 1. The numbers of status conservative sites in the positive and negative datasets.

peptide length	class	position 1		position 2		position 3		position 4		position 5		position 6		position 7		position 8		position 9		position 10	
		no	yes	no	yes	no	yes	no	yes	no	yes	no	yes	no	yes	no	yes	no	yes	no	yes
11	neg	1846	63	1843	66	1839	70	1835	74	1834	75	1834	75	1834	75	1834	75	1834	75	1834	75
	pos	1805	104	1800	109	1795	114	1788	121	1785	124	1785	124	1785	124	1785	124	1785	124	1785	124
13	neg	1791	86	1785	92	1785	92	1779	98	1776	101	1773	104	1773	104	1773	104	1773	104	1773	104
	pos	1753	124	1747	130	1745	132	1737	140	1729	148	1727	150	1727	150	1727	150	1727	150	1727	150
15	neg	1739	134	1727	146	1722	151	1711	162	1702	171	1697	176	1692	181	1692	181	1692	181	1692	181
	pos	1711	162	1702	171	1691	182	1682	191	1671	202	1666	207	1660	213	1660	213	1660	213	1660	213
17	neg	1677	192	1659	210	1644	225	1626	243	1612	257	1605	264	1593	276	1590	279	1590	279	1590	279
	pos	1665	204	1648	221	1640	229	1627	242	1611	258	1603	266	1597	272	1594	275	1594	275	1594	275
19	neg	1629	246	1602	273	1579	296	1556	319	1540	335	1531	344	1516	359	1510	365	1507	368	1507	368
	pos	1615	260	1589	286	1569	306	1552	323	1538	337	1527	348	1521	354	1513	362	1506	369	1506	369
21	neg	1577	295	1534	338	1503	369	1472	400	1450	422	1436	436	1419	453	1406	466	1399	473	1393	479
	pos	1549	323	1520	352	1494	378	1468	404	1450	422	1427	445	1412	460	1401	471	1394	478	1392	480
23	neg	1513	349	1468	394	1434	428	1394	468	1371	491	1354	508	1327	535	1307	555	1297	565	1287	575
	pos	1475	387	1434	428	1405	457	1370	492	1343	519	1314	548	1300	562	1291	571	1276	586	1269	593
25	neg	1461	407	1403	465	1360	508	1317	551	1295	573	1270	598	1246	622	1224	644	1211	657	1197	671
	pos	1441	427	1390	478	1360	508	1330	538	1294	574	1263	605	1236	632	1217	651	1202	666	1194	674
27	neg	1426	441	1366	501	1319	548	1266	601	1235	632	1209	658	1182	685	1164	703	1150	717	1133	734
	pos	1394	473	1335	532	1290	577	1250	617	1207	660	1173	694	1149	718	1132	735	1114	753	1104	763
29	neg	1399	480	1320	559	1268	611	1212	667	1172	707	1140	739	1108	771	1082	797	1065	814	1046	833
	pos	1375	504	1305	574	1250	629	1210	669	1161	718	1127	752	1103	776	1085	794	1061	818	1046	833

peptide length: the residue window as the query peptide for BLAST research
position: alignment positions to calculate the status conservation
yes: the number of conservative sites
no: the number of non-conservative sites
neg: negative dataset
pos: positive dataset

Supplementary Table 2. The p-values of status conservation between the positive and negative datasets.

peptide length	position 1	position 2	position 3	position 4	position 5	position 6	position 7	position 8	position 9	position 10
11	0.00155	0.00115	0.00116	0.00072	0.00047	0.00047	0.00047	0.00047	0.00047	0.00047
13	0.00859	0.01046	0.00721	0.00603	0.00255	0.00345	0.00345	0.00345	0.00345	0.00345
15	0.10199	0.15886	0.08501	0.11737	0.10164	0.10569	0.09874	0.09874	0.09874	0.09874
17	0.55881	0.60857	0.88060	1.00000	1.00000	0.96260	0.88967	0.89016	0.89016	0.89016
19	0.53436	0.58218	0.68890	0.89652	0.96604	0.89950	0.86780	0.93416	1.00000	1.00000
21	0.23459	0.58372	0.74355	0.90496	1.00000	0.75792	0.81937	0.88004	0.88063	1.00000
23	0.12787	0.19228	0.28105	0.38888	0.31965	0.15622	0.34997	0.59252	0.47819	0.54823
25	0.45537	0.65130	1.00000	0.66573	1.00000	0.83360	0.75518	0.83658	0.78433	0.94565
27	0.23803	0.27243	0.31794	0.60056	0.35297	0.23335	0.27957	0.29717	0.24106	0.34980
29	0.39343	0.61873	0.55534	0.97281	0.73671	0.68906	0.89452	0.94735	0.92135	1.00000

peptide length: the residue window as the query peptide for BLAST research
position: alignment positions to calculate the status conservation

Supplementary Table 3. Validation of whether the "polymorphic but not associated with diseases" rule for the negative sites was reasonable.

Model	Random Forest	BayesNet	Logistic	Multilayer Perceptron
Predicted positive sites*	13883	13772	14749	14878
Predicted positive sites passed the rule	499	501	512	566
p-value (chi-squared test)	1.12E-04	2.95E-04	3.55E-06	4.47E-03

Total high-quality phosphosites: 82818; sites passed the rule: 3595.
*false positive rate = 0.1
